# When Employees are Emotionally Exhausted Due to Abusive Supervision. A Conservation-of-Resources Perspective

**DOI:** 10.3390/ijerph16183300

**Published:** 2019-09-08

**Authors:** Zubair Akram, Yan Li, Umair Akram

**Affiliations:** 1 School of Management and Economics, Beijing Institute of Technology, Beijing 100081, China; 2 Guanghua School of Management, Peking University, Beijing 100871, China

**Keywords:** abusive supervision, emotional exhaustion, job demands, counterproductive work behavior, conservation of resources theory, China

## Abstract

This study represents an important step towards understanding why supervisors behave abusively towards their subordinates. Building on the conservation of resources theory, this study investigates the impact of abusive supervision on counterproductive work behaviors (CWBs) from a stress perspective. Furthermore, job demands play a significant moderating effect, and emotional exhaustion has a mediating effect on the relationship between abusive supervision and CWBs. A time-lagged design was utilized to collect the data and a total of 350 supervisors-subordinates’ dyads are collected from Chinese manufacturing firms. The findings indicate that subordinates’ emotional exhaustion mediates the relationship between abusive supervision and CWBs only when subordinates are involved in a high frequency of job demands. Additionally, emotional exhaustion and abusive supervision were significantly moderated by job demands. However, the extant literature has provided that abusive supervision has detrimental effects on employees work behavior. The findings of this study provide new empirical and theoretical insights into the stress perspectives. Finally, implications for managers and related theories are discussed, along with the boundaries and future opportunities of this study.

## 1. Introduction

Over the past decades, counterproductive work behaviors (CWBs) have become an increasingly popular topic of the study among organizational researchers [1,2,3,4,5,6]. CWBs involve activities that are unhealthy for the effectiveness of the organization. These activities slow down the normal working of the organization. Different studies have shown the economic effects of CWBs on organizations. Recent works have indicated that global businesses suffer losses of around US$2.9 trillion annually due to fraudulent activities [1].

Interpersonal relationships are developed in the work environment with none being of more importance than those that the employees have with the manager or supervision to whom they report [7,8]. In reviewing past research there has been the discovery of empirical evidence revealing that supervisor abuse has a relationship to the employees’ workplace deviance while also identifying some of the situational contexts which can aid in building a much better understanding concerning when as well as why employees’ workplace deviance is less likely to occur [9]. However, it is of importance to realize that empirical research explores the boundary effects, with the information being somewhat limited. This entails a discussion of the impact of abusive supervision and counterproductive work behavior with a moderated mediation model that can aid in the correction of this adverse action.

The primary concern relative to this study is to discover how abusive supervision impacts workplace deviance behavior [10]. All organizations and businesses must have an understanding of the importance of employees and leaders working together in order to aid the business in growing while also gaining a competitive advantage in the market, wherein human capital is being of great importance. However, when abusive supervision and counterproductive work behavior are present in the workplace, the company can become inefficient as well as non-productive, which are actions that will affect customer service as well as the products created by the company [3].

Emotional exhaustion is characterized by feelings of being drained, overstretched and depleted of one’s resources [11]. The limitation of personal resources causes employees to experience emotional exhaustion (EE). EE is a state of physical and psychological depletion [5], and is one of the most dysfunctional attitudes in the contemporary high stress work environment. Emotional exhaustion refers to behavior that can distract oneself emotionally and cognitively from work [11]. Abusive supervision is responsible for employees’ emotional exhaustion and ultimately supports employees to engage in counterproductive work behavior [12,13]. Employees who have faced abusive supervision have experienced emotional exhaustion that has, in turn, led to counterproductive work behaviors [14]. Emotional exhaustion can be conceptualized as a loss of resources that are essential for the completion of work [15], Conversation of Resources (COR) theory [15], is related to emotional exhaustion. According to COR theory, people struggle in order to retain, protect, and build upon that which they value (i.e., resources). Emotional exhaustion occurs when these valuable resources are lost, or when people are unable to get expected returns. From the perspective of COR theory, it can be a useful viewpoint from which to elaborate on how CWB influenced by abusive supervision [15]. In the light of COR theory, the current research investigates the influence of abusive supervision, a type of workplace stressor, on CWB. The subordinates of abusive supervisors are more likely to perceive emotional demand and a lack of resources during their interactions—this leads to emotional exhaustion.

Job demand can be defined as the psychological, physical, organizational, and social aspects of the job that requires sustained psychological/physical (emotional or cognitive) skills or efforts [16]. Therefore, this is related to specific psychological costs [16]. Employees working under abusive supervision may decide to engage in CWB because they anticipate that they will not get sufficient resources when the job demands are high. High job demand and control all of the necessary resources by an abusive supervisor create emotional exhaustion among subordinates. Subordinates working under high job demands require considerable resources for the completion of tasks [16,17]. However, prior study has indicated that better resource management abilities means that subordinates will experience fewer undesirable attitudes (i.e., turnover intentions, emotional exhaustion, and dissatisfaction), in that those who work under abusive supervision will not perform as well as those who capably manage their resources [18]. Therefore, this study considered job demands as a moderator.

Today, more than ever, organizations are placing a focus on the reduction of counterproductive work behavior as these behaviors lead to more difficulties within the workplace, creating, at best, a lethal environment for dissension, confusion, and conflict within the workplace [19]. There will also be less productivity and efficiency, thereby increasing the cost of products and services. Therefore, this study has been performed to share a moderated mediation model which can be used to identify factors to overcome counterproductive work behavior, thereby creating a peaceful work environment for all employees.

Using a moderated mediation framework, we proffered that emotional exhaustion play a significant mediating role between CWB, and that abusive supervision differs depending on job demands. Because job demands are always associated with subordinate’s emotional exhaustion, high job demands are known to intensify the significant and positive effect of abusive supervision. According to our study, the detrimental effects of abusive supervision on emotional exhaustion increases with job demands, and a positive indirect effect between CWB and abusive supervision is strengthened.

Therefore, the current study aims to answer the following three questions. In what way and under what conditions does abusive supervision lead to CWB? Does abusive supervision facilitate CWB, and if so, what are the underlying mechanisms and how do job demands influence these associations? How and when are employees emotionally exhausted due to abusive supervision and CWB? By focusing on these issues, present study creates new insights that organization can employ to alleviate the harmful effects of abusive supervision rather extend the existing literature.

More specifically, the significant contribution of the present study is to test and develop the theoretical model by applying the conservation of resources (COR) theory of stress developed by [15]. For this study, manufacturing firms were selected in China. There is a need to conceptualize abusive supervision as workplace stressors that cause employees to lose valuable personal resources. Abusive supervision and CWBs relationship have been explained by the COR theory to demonstrate the stress process. Therefore, the research provides several important contributions with respect to abusive supervision and COR Theory. First, the present study contributes to the existing research of abusive supervision because this factor is so harmful for employees and organizations. Many supervisors habitually abuse their employees with the organization’s best interests in mind. Furthermore, the context of this study is entirely different than previous conducted studies. Second, we extend the current literature review of abusive supervision through an understanding of the role of employee’s job demands to classify the limitations of connections between emotional exhaustion and abusive supervision, which has not been previously established with this model. Thus, the mediator, and moderating role of job demands and emotional exhaustion, is yet another contribution of this study. Third, by employing the moderated mediation model of Edwards and Lambert [20], the present study discovers the circumstances under which abusive supervision is associated with CWBs. The primary focus of existing literature on abusive supervision conclude and recipients that abusive supervision is always harmful and costly [12,21,22]. The context of this study is another contribution of this study.

## 2. Theoretical Underpinning & Hypotheses Development

### 2.1. Conservation of Resources Theory

The conservation of resources theory explains that individuals attempt to get, sustain and preserve the resources such as energy and time [15]. Theoretical clarification for whether and what situations interpersonal mistreatment behavior through emotional exhaustion is suggested by emotional exhaustion. COR theory proposes that individuals are influenced to protect their current personal resources and get new resources to achieve their goals. However, when these resources are lost or threatened, individuals suffer consequent emotional exhaustion and psychological arousal [15,22]. Psychological stress arises among individuals when they are threatened, feeling loss, and have no power to regain resources. Subordinates experienced the loss of valuable resources due to the aggressive behavior of the supervisor. Generally, Abusive behaviors categorize into different types such as belligerent eye, shouting, threat employees for job loss, could cause abused employees to perceive the loss of control. Control over a job is a significant resource available to the employee. Therefore, abused employees suffer frustration due to losing control over valuable resources and personal autonomy. In response to this frustration and losing control, subordinates get involved in counterproductive work behavior [23].

### 2.2. Abusive Leadership and Workplace Deviance Behavior

Part of leadership responsibility concerns giving focus to the positive behavior of the employees in an attempt to recognize harmful behavior, bringing an end to that behavior before it spreads within the department and the organization. This is of great interest when viewing the leader as it is the responsibility of the leader to lead by example. Leaders and managers also focus on the positive aspects of behavior as this behavior is of benefit to the organization. However, the subject of abusive supervision has caused management to change their focus with the examination of abusive supervision and the results of counterproductive work behaviors [24,25,26].

When viewing the subject of negative behaviors, there is the need to focus on the interpersonal matters, to include the manager being disrespectful while also being abusive and talking to others in an abusive tone. Research also revealed results that these negative behaviors bring about the negative impact on the employee’s mental health as well as a decrease in efficiency while reducing job satisfaction of employees [26]. Several studies have been performed in the past to identify the effects between deviant behavior and abusive supervision with the studies revealing that bad supervision is correlated to workplace deviance behavior by the employees [9].

### 2.3. Abusive Supervision as a Workplace Stressor

The conceptualization of abusive supervision, according to Tepper [8], can be described “as [a] subordinate’s perception of the extent to which supervisors engaged in sustained hostile verbal and non-verbal behaviors, excluding physical contact”. Furthermore, according to Tepper, [10] abusive supervision can be measured by encouraging employees to report the frequency at which their immediate supervisors perform numerous hostile acts (e.g., the reason of humiliation, ideas are ludicrous and insulting employees in front of others). An essential feature of this conceptual definition, one that has been a source of misplaced criticism and confusion, has to do with the characterization of abusive supervision as a subjective perception. The research of abusive supervision has shown its harmful for organizations. Previous studies have shown the negative association between organizational citizenship behavior (OCB) and abusive supervision, employee’s voice behavior, and organizational commitment [12,27], and positively related to the deviant, resistant, and aggressive behaviors of employees [3].

### 2.4. Counterproductive Work Behavior (CWBs)

“CWBs, as behaviour intended by employees, is harmful to the legitimate interest of an organization” [1,28]. CWBs is one of the undesirable responses of employees to abusive supervision [29]. Typically, CWBs including deliberately misusing money, reducing output, showing offensive acts, hindering other colleagues, disobeying orders, and even theft resources. These types of behavior refer to a set of distinct acts that exhibit the following features: they are volitional (as opposed to mandated or accidental) and harm organizations and their stakeholders, such as coworkers, clients, supervisors and customers [30]. According to Spector and Fox [30], CWB has two different dimensions: passive withdrawal and active deviant behavior. Passive withdrawal involves an attempt to avoid activities such as cyber loafing and daydreamin whereas active deviant behavior is the active involvement in damaging organizations (e.g., aggression, sabotage, and theft).

### 2.5. Perception of Abusive Supervision and CWBs

According to Tepper, [8], CWBs are related to a subordinate’s perception of the extent to which supervisors are engaged in sustained of hostile verbal and non-verbal behaviors excluding physical contact”. Cropanzano and Mitchell [31] have used social exchange theory to define the rules and norms of exchange and the nature of the relationship between supervisors and subordinates. Social exchange theory emphasizes the principle of reciprocity. A relationship evolves over time to become one of trust, loyalty and mutual communication. In such relationships sacrifices by one party oblige the other to respond in kind. Based on this theory, argue that when an individual feel misguided by their supervisor, they retaliate through negative reciprocity. In other words, the abusive behavior of the supervisor prompts subordinates to engage in aggressive behavior.

Such dynamics are reflected in other studies. For instance, reciprocal exchange occurs when two parties are involved in a give-and-take relationship in regard to resources. The abusive behavior of a supervisors urges subordinates to breach their psychological contract and engage in counterproductive work behavior-hindering the productivity of an organizations [1]. Consistent with Zhang & Bednall, [32], have demonstrated that the anger of employees will increase as a result of the abusive behavior of employers. According to this argument, the devious and unproductive acts adopted by subordinates are a direct response to abusive leadership [33]. Employees intentionally engage in harmful action towards organization when they find their supervisor abusive.

Unfortunately, most of the studies to date on employees’ reactions to abusive supervision have focused on supervisor-targeted behaviors or CWB in general [34], resulting in organization-targeted outcomes (CWB-O) remaining unclear. In addition, the association between abusive supervision-CWB-O may be complex. For instance, employees’ reactions may not be proportionate to the degree of abuse. Moreover, it has been recommended that initial retaliations to abusive supervision may be relatively mild (e.g., withdrawal), although later escalate to more severe behaviors (e.g., destructions of property) as the abuse continues [34]. This indicates a nonlinear component in the association between CWB-O and abusive supervision. Based on the aforementioned, we predict a positive relationship between abusive supervision and CWB as a response to the behavior of supervisors and directed toward the organization. Thus, we hypothesized

**Hypotheses 1** **(H1):** The positive relationship between abusive supervision and CWBs.

### 2.6. Mediating Effect of Emotional Exhaustion

Many researchers have found that abusive supervision is associated with emotional exhaustion as a work place stressor [10,35]. According to Maslach and Leiter [36] emotional exhaustion is a symptom of psychological strain and can be defined as feeling of being emotionally exhausted in terms of one’s physical and emotional resources. Research indicates that emotional exhaustion leads to interpersonal dysfunctional in the workplace, including CWBs. For instance, according to Jahanzeb and Fatima [37], employees who experience significant emotional exhaustion tend to be engaged in deviant behavior in their workplace. Deviant behaviors involve intentionally damaging and organization’s property, as well as reducing the time spent working and increasing that spent on personal tasks [27]. Employees experience emotional exhaustion due to interpersonal conflict between themselves and abusive supervisors, as well as with other abused subordinates. The intended attack on a subordinate’s self-efficacy and self-esteem by an abusive supervisor may result in their emotional exhaustion [38,39]. As such, employees under abusive supervision are more likely to experience emotional exhaustion and are thus more likely to engage in negative destructive workplace behavior.

Mistreating behavior of abusive supervision is likely to experience higher levels of emotional exhaustion among subordinates at work. Employees also experience emotional exhaustion when essential resources are unavailable as a result of an abusive supervisor. Subordinates may engage in CWB when they feel unable to minimize the loss of valuable resources [39]. Emotional exhaustion urges abused employees to engage in counterproductive work behaviors. Depletion of resources by the abusive leader places the employee in the state of emotional exhaustion and demonstration of CWB. Therefore, drawing insight from the COR theory, the current study investigates the mediating role of emotional exhaustion between counterproductive work behavior and abusive supervision.

Previous research was performed to examine the relationship that exists between the subordinates’ essential or central self-evaluations and the supervisors’ abusive supervision [40]. The researchers also examined whether or not the subordinates perceived the coworker support, as well as the subordinates’ weakness to emotional contagion, was moderated by the relationship that existed between the supervisors’ abusive supervision and the subordinates’ emotional exhaustion [41]. The findings revealed that the core self-evaluations were damagingly associated by abusive supervision, while the abusive supervision was found to be positively related to the emotional exhaustion [14]. The perceived coworker support, as well as susceptibility to the emotional contagion, could be moderated when viewing the relationship that existed between the abusive supervision and the emotional exhaustion [40].

Emotional exhaustion can be conceptualized as the loss of resources necessary to respond to work demands. Emotionally exhausted employees may engage in CWB in efforts to protect limited resources. CWB involves intentionally damaging organization property, as well as reducing the time spent working and increasing that spent on personal tasks [27]—these acts of CWBs may be a misguided attempt to rebuild resources by investing energy into “lashing out” against the organization as a way to gain a sense of control over the source of resource loss [27].

Mental exhaustion and low energy may be experienced by individuals who work under pressure, while psychological stress may occur due to the depletion of resources. The resulting emotional exhaustion reduces and individual’s ability to cope with and meet the emotional demands at workplace [42]. Therefore, we expect that subordinates who are subject to abusive supervisory behavior are likely to display emotional exhaustion at the work-place and argue for a positive relationship between abusive supervision and emotional exhaustion. Thus, we hypothesized:

**Hypotheses 2** **(H2):** The positive relationship between abusive supervision and CWBs is mediated by emotional exhaustion.

### 2.7. Job Demands as Moderator

One salient and resource-relevant situational characteristics is job demands, defined as “psychological, physical, organizational and social aspects of job that required sustained psychological/physical (emotional or cognitive) skills and therefore is related to certain psychological costs [16] (p. 312)”. Given that resources are central to both demands and exhausted. According to the COR theory, as in the study of Halbesleben [43], principles of primacy loss, damage of lose resources outweighed the advantage of obtaining resources. In our model, high job demands represent such contexts which makes role of abusive supervision for resources stresses more silent to supervisors.

An employee with high job demands from an abusive supervisor will experience high emotional exhaustion. The moderating effect of job demands supporting by COR theory recommends that lack of possession of resources from abusive supervisor lead subordinates to high emotional exhaustion [44]. High job demands convey message to subordinates about abusive supervisor behavior that he is not in force to provide sufficient resources to subordinates for making job done. This pressure from an abusive supervisor will lead the subordinate to experience emotional exhaustion.

**Hypotheses 3a** **(H3a):** *Job demands moderates the positive relationship between abusive supervision and emotional exhaustion such that the relationship is stronger when job demands are high.*

The effect of emotional exhaustion on CWB will positively strength with the interaction between abusive supervisor and job demand. It can be defined as employee with high job demand, will experience high emotional exhaustion due to the abusive behavior of the supervisor not releasing a necessary resource that are required for completion of the job. Subordinates working under high job demands are required to allocate high levels of energy and attention to complete a big task in a short time, and meeting these demands requires considerable resources [16,45,46]. This will create stress for subordinate and involving in counterproductive behavior.

So, it is concluded that when an employee experiences abusive supervision and high job demands, they will face high emotional exhaustion, and this will lead them to engage in CWB. Overall, we suggest that a positively association between abusive supervision with emotional exhaustion will be stronger when job demand is high. Moreover, integrating these arguments and the proposition that abusive supervision has a positive indirect effect on counterproductive work behavior (CWBs) via emotional exhaustion, we propose that job demands by the indirect effect.

**Figure 1:**
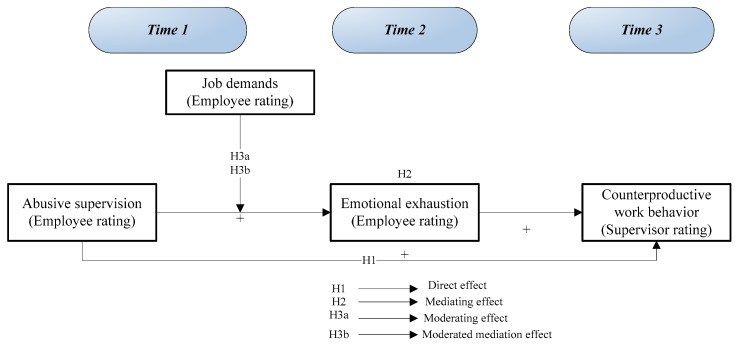
A moderated-mediation model.

According to the results of the aforementioned studies, the scholars, therefore, believe that the framework conforms to the first-stage moderation model is developed by Edwards and Lambert [20]. In this model, (abusive supervision) is independent variable, moderator (job demands), both interactively influences on (emotional exhaustion) as a mediator, thereby exerting influences on (counterproductive work behavior) is dependent variable. Figure 1 shows the graphical representation of the all variables relationship.

**Hypotheses 3b** **(H3b):** *Job demands moderates the positive indirect relationship of abusive supervision on CWB* via *emotional exhaustion such that the indirect relationship is stronger when job demands are high.*

## 3. Methods

### 3.1. Sample Procedures

Data were collected from three Chinese manufacturing firms located in an eastern province of China. With the approval and assistance of senior management, we randomly distributed 500 questionnaires of selected companies. The pool of respondents comprised both employees and supervisors.

The data were collected through a three-waves survey, which is likely to minimize the common method bias [47]. In the first-wave survey (time1), employees rated their leaders’ abusive supervision, job demands, control variables, and provided demographic information. One month after the first, second wave survey was conducted (time2), employees rated their emotional exhaustion during the previous month. Finally, in the third-wave survey (time3)*,* being conducted one month after the second-wave survey, supervisors were asked to evaluate the counterproductive work behaviors of their subordinates in the previous month.

Two members from the research team explained the purpose of the study and data collection procedure for both supervisors and employees during the working hours. All of the participants received a cover letter outlining related to study, assurance of anonymity, voluntary nature of participation, and then the participants returned the envelope and questionnaires. During the first and second wave (time1) and (time2), the data were matched to the responses of the supervisors collected during the third wave (time3), using a coding system based on information provided by the managers.

A total of 425 questionnaires were obtained from employees during the first-wave survey. After one month, the second wave-survey collected 400 questionnaires from the employees. The response rate of the first two surveys was 85%. The third-wave survey was distributed to the employees and supervisors. We received 350 completed questionnaires from supervisors and employees, yielding a response rate of 70%. Taken together, our final sample comprised was 350 supervisors-subordinates’ dyads. From employee, we received 350 completed self-reported questionnaires and 40 from supervisors. Thus, yielding a response rate of 70%. Under one supervisor, 8–10 employees were working, so one supervisor was responsible for completing the questionnaire for 8–10 employees.

Of the 350 employees selected for this study, 85% were male, who had worked for the organization for 9.38 years (Standard Deviation (SD) = 0.96), and had an average age of 32.43 years (SD = 6.52). The position in the organizations frontline employees were 78.8%, managers of frontline were 13.9%, and middle-level managers were 7.3%. Of the 40 supervisors, 60% were male, their average age was 42.64 years (SD = 5.68), and their average tenure was 8.39 years (SD = 7.27).

### 3.2. Measures

All scales were originally developed in English. The target respondents of this study were Chinese people. We back-translate the questionnaires [48]. First, questionnaire was translated into Chinese and then back translation into English with the help of two post-graduate students who are majors in foreign linguistics and two post-doctoral researchers. Thirty Ph.D students were recruited for pilot survey. After the pilot survey, recommendations and suggestions were collected and to revise the questionnaire before the final survey. All response options ranged from 1 (strongly disagree) to 5 (strongly agree).

#### 3.2.1. Job Demands

This variable using an eight-items scales are developed by Van Veldhoven and Meijiman [49]. An example of sample items is included “I have to work extra hard to finish a task”. The scale is reliable (Cronbach’s α = 0.86).

#### 3.2.2. Emotional Exhaustion

Five items were used to evaluate the emotional exhaustion developed by Maslack burnout inventory scale, Maslack, Jackson & Leiter’s [50]. One sample item is included as “I feel burnout of my work” (Cronbach’s α = 0.90).

#### 3.2.3. Abusive Supervision

In order to assess the abusive supervision used five items were developed by Tepper [8]. This variable scale ranges from 1 = (never) to 5 = (very often). An example of one item is “My supervisor tells me my thoughts or felling are stupid”. (Cronbach’s α = 0.89).

#### 3.2.4. Counterproductive Work Behavior

CWB used eight-items scale is adopted from Dalal and Welch [3]. A sample item is included as “Behaved in an unfriendly manner”. (Cronbach’s α = 0.96).

#### 3.2.5. Control Variables

Demographic characteristics such as employee’s education, gender, age, tenure, level of organization. For supervisors, education, gender, age, level of organization, and tenure. 20 items of Waston and Tellegen [51] related to positive and negative effects used in present study. The employees show that how they normally feel in terms of 10 positive affect (e.g., joyful, interested, and satisfied) and 10 negative effects (e.g., irritated, nervous, disappointed) used by five response options from (1 = Not at all, 5 = Extremely). The alpha value for positive affect (PA) (Cronbach’s α = 0.91) and for negative affect (NA) (Cronbach’s α = 0.94).

## 4. Results

### 4.1. Attrition Analysis

We collected the data from employee’s different time waves, following the method of Goodman and Blum [52] to test the first and second wave surveys of data collection. First, multiple logistic regression was used to examine the time survey as an endogenous variable, and employees’ education, age, organizational level, tenure, gender, abusive supervision and job demands as independent variables. The non-significant regression coefficient was observed through our results. T-test was conducted that showing there is significant difference in the mean values of employees, education, age, tenure, organizational level, gender abusive supervision and job demands between time one and two. Significant mean differences between these variables are evidenced by these results. Employees drop out randomly as suggested by results.

### 4.2. Confirmatory Factor Analysis (CFA)

CFA analysis is conducted to measure the all key variables (abusive supervision, emotional exhaustion, job demands, and counterproductive work behavior) in this study. Present study used the discriminant and convergent validity of the four variables. We used LISREL 8.80 (Educational Testing Service in Princeton, New Jersey, USA) to conduct a series of confirmatory factor analysis (CFA). According to our CFA results, the items with the lowest and highest loadings, all the items were assigned to one indicator. Therefore, we computed the mean of the scores of items and scores of each indicator. CFA Results reported as Table 1, we hypothesized four-factor model fits the data well, with X^2^(80) = 129.16, *p* < 0.05; Tucker-Lewis index (TLI) = 0.94; confirmatory fit index (CFI) = 0.96; and root mean square error of approximation (RMSEA) = 0.046. Furthermore, all items of the variable were assigned to one of the respective indicator variable and factor loadings of all items are greater than 0.60 which support the convergent validity.

Further, the discriminant validity of the four key constructs in our study were examined by comparing the four-factor model against alternative models. As reported in Table 1, the model comparison results indicate that the four-factor model fit the data better than the one-factor model and three-factor models by comparing the fit indices and X^2^ change [53]. Therefore, the discriminant validity was well achieved in our study.

### 4.3. Descriptive Statistics

Mean, standard deviation, Pearson zero-order correlation, and reliabilities among all study variables are reported in Table 2. The reliabilities of all variables meet the threshold value 0.70. Table 2 presented, abusive supervision is positively associated to emotional exhaustion (*r* = 0.22, *p* < 0.01) and positively correlated to CWB (*r* = 0.13, *p* < 0.01). Furthermore, emotional exhaustion is positively correlated with CWB (*r* = 0.18, *p* < 0.01). These results are consistent with and provide initial support for our hypotheses.

### 4.4. Hypotheses Testing

Hierarchical multiple regression analyses were conducted to test our research hypotheses. According to the Hypothesis 1, Table 3 indicated that abusive supervision is positively associated with counterproductive work behavior (*β* = 0.11, *p* < 0.01; model 3). Therefore, H1 was supported.

Hypothesis 2 follow the conditions of Baron and Kenny [54], relationship between abusive supervision and counterproductive work behavior is positively mediated by emotional exhaustion. In order to test H2, all control variables entered (education, age, gender, tenure and organizational level), followed by exogenous variables (abusive supervision) in the second step, and emotional exhaustion was entered in third step as mediator variable. The results shown in Table 3 evince that (i) abusive supervision is positively related with CWB (*β* = 0.11, *p* < 0.01; model 3), (ii) abusive supervision is also positively related to emotional exhaustion (*β* = 0.22, *p* < 0.01; model 3), (iii) emotional exhaustion is positively related to CWB (*β* = 0.18, *p* < 0.01; model 4) (iv) in this step when we entered emotional exhaustion, the association between abusive supervision and counterproductive work behavior is nonsignificant (*β* = 0.23, n.s. model 4). The relationship between emotional exhaustion and CWB is still positive with (*β* = 0.27, *p* < 0.01 model 4). According to the results emotional exhaustion mediates the relationship between abusive supervision and CWB. Hence, H2 was supported.

**Figure 2:**
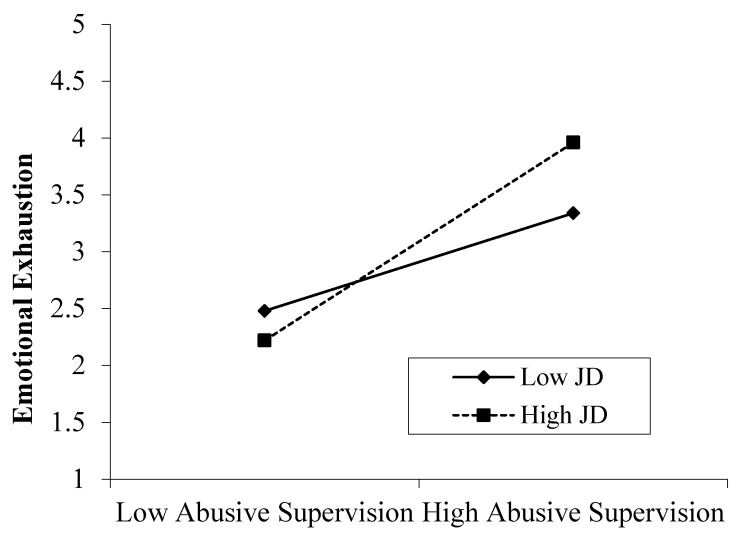
Moderating effect of abusive supervision and job demands (JD) on emotional exhaustion.

Hypothesis 3a reports that job demands moderate the relationship between abusive supervision and emotional exhaustion. In order to test the H3a, Four-steps were employed for hierarchical moderated multiple regression analysis. The control variables, exogenous variable (abusive supervision), moderating variable (job demands), and finally, the interaction between abusive supervision and job demands, were entered in separate steps. The interaction between abusive supervision and job demands was positively associated with emotional exhaustion (*β* = 0.29, *p* < 0.01, model 2). Table 3 indicates that 3% of variance was found in emotional exhaustion (∆R2 = 0.03, ∆F = 8.03, *p* < 0.01). The interaction pattern is shown in Figure 2. In order to illustrate the relationship, the following recommendations of Aiken and West [55], revealed one standard deviation above and below the mean of job demands. Consequently, Hypotheses 3a was supported.

Finally, we used method of moderated path analysis following Edwards & Lambert [20] to test Hypothesis H3b. Table 4 indicated the results of moderated analysis (H3b). The indirect effect of abusive supervision on counterproductive work behavior (CWB) via emotional exhaustion for lower levels of job demands (*β* = 0.02, 95% CI (0.08, 0.29) n.s.) and strengthen the high-level job demands (*β =* 0.08, *p* < 0.01, 99.5% CI (0.04, 0.12)); and the difference of high and low levels of job demands was significant (95% CI (0.02, 0.11), *β* = 0.06, *p* < 0.01).

## 5. Discussion and Implications

### 5.1. Discussion

A number of latest researches confirmed that abusive supervision is strongly related with employees’ dysfunctional or negative behaviors and attitudes [12,56]. We tested and developed a model from a stress perspective that explains how and when in abusive supervision is related to CWB. The finding for the study supports the positive relationship between abusive supervision and CWB. Reason of subordinate’s involvement in counterproductive work behavior is supervisor abusive behavior towards subordinates. A lack of social support and well-being from supervisor motivate subordinates to involve in counterproductive work behavior. The supervisor who fails to give complete guideline to subordinates about work; instead this humiliates them urge subordinates to engage in counterproductive work behavior. Findings of our study is coherent with [57].

The findings of our study indicate that emotional exhaustion mediates the association between CWB and abusive supervision. An employee will experience emotional exhaustion due to interpersonal conflict between abusive supervisor and abused subordinates. A noxious environmental engenders the abusive supervisor and endorses feelings of feebleness and loss of control in victims. According to COR theory, the threat by the abusive supervisor for releasing resources to subordinates will increase emotional exhaustion and lead to the CWB [58]. Imposing excessive emotional demands on subordinates’ resources, retard them to regain resources through ethical means and urge them to involve in counterproductive work behavior. Results are integrated with the study of [59].

The moderating effect of job demand on the relationship between abusive supervision and emotional exhaustion is of concern. This study shows that an association between CWB and abusive supervision is reliant on emotional exhaustion. Mediation moderation analysis result exhibits that abusive supervisor is positively linked with emotional exhaustion and make an impact on CWB to a more considerable degree for those who have high than low job demand. This process arises because of high job demand from abusive supervisor create pressure and experience high emotional exhaustion. High job demand leads to employee involvement in CWB for the fulfillment of the task by using unpleasant mean to gain resources.

When viewing the workplace, the role of the supervisor plays a vital role to increase or decrease employee efforts toward task accomplishment. According to COR theory, the research tries to explore the relationship between abusive supervision and counterproductive work behavior. It also investigates the mediating effect of emotional exhaustion on the relationship of abusive supervisor and counterproductive work behavior moderated by job demand. As abusive supervision increases, subordinates suffer from emotional exhaustion which urges employee involvement in counterproductive work behavior. This stress will increase when job demand is high at the behest of an abusive supervisor.

### 5.2. Implications

#### 5.2.1. Implication for Theory

Our research makes different key contributions to abusive supervision and conservation of resources (COR) literature. Although a large number of studies have applied COR theory in organizational behavior research [15]. First, the research identifies how emotionally exhausted subordinates due to abusive leader engage in CWB. Therefore, the mediating role of a subordinate’s emotional exhaustion is being tested with the relationship as mentioned above. The conceptualization of subordinates’ emotional exhaustion as a mediator may result from experiencing high stress, as a response to an abusive supervisor, encouraging them to engage in CWB. Results may provide insight to understand how abusive supervisor effects subordinates’ emotions and feelings. This is consistent with COR theory; our findings show that abusive supervision indirectly affects CWB through emotional exhaustion [22].

Second, our research contributes to the abusive supervisory literature by showing when abusive supervisory behavior is less likely (in terms of emotional exhaustion and CWB) for supervisors. While previous studies have established the impacts of abusive supervisory behavior on employees [14], and the impacts of engaging in abusive supervisory behavior for supervisors themselves has been largely overlooked [59]. More specifically, abusive behavior is more beneficial when supervisors high (vs. low) job demands. Furthermore, replenishing effects and resource conservation of abusive supervisory behavior also hinges on how stressful or demanding supervisors’ jobs. We found that the beneficial effects of abusive supervisory behavior on exhaustion and CWB would be strengthened for supervisors confronted with high (vs. low) job demands. Finally, abusive supervision may be one means through which supervisors converse their limited resources and maintain a high level of exhaustion and sense of control in stressful situations [27].

Third, research on abusive supervision and subordinates’ emotional exhaustion is expanded through investigation the strongest indirect effect of job demands. Experience of emotional exhaustion will be fewer when job demand is low. Findings suggest individual differences in job demands and more attention is required since they can help elucidate the effects of abusive supervisory behavior on subordinates’ emotional exhaustion and ultimately on their work. Our study findings suggest that job demands play a contingent role of abusive supervision and CWB relationship through emotional exhaustion.

#### 5.2.2. Implications for Practice

The results indicate that subordinates’ emotional exhaustion and subsequently CWBs will increase by abusive supervisor. Strict actions like Internal disciplinary and legal system should be applied to eradicate the adverse effects of abusive supervisory. Further, organizations should carefully observe activities of managers who are involved in aggressive actions to abused employees, must be considered these harsh behaviors at the time of promotion of managerial positions. Furthermore, the training session should be conducted at the supervisory level to train supervisors of changing their attitudes and behaviors towards an employee in order to reduce stress and provide them a healthy environment.

Second, organizations must give proper guidelines to supervisors for undistorted employee CWB. It is proved from current findings and past evidence that the relationship between abusive supervision and deviance behavior is bidirectional e.g., [60]. In order to cope with employee deviance work behavior, the organization should identify effective ways. As an example, ethical leadership behavior helps the organization to implement an ethical code of conduct morally manage employee’s behavior. By following the study of Brown et al., [61] a leader must act as a role model to ethically guide employees for ethical behavior.

Another implication of our study found that job demands moderate the positive relationship between abusive supervision and emotional exhaustion, such that this relationship existed only when perceived job demands were high (vs. low). Under these circumstances, supervisors may experience sometimes have been exhausted by abusive subordinates; it provides them a sense of control and free from the resource-consuming process of suppressing aggression. Some ways of reducing abusive supervisors’ perceived job demands might include reducing their workload, training them to work more efficiently and providing them to more resources. For instance, senior level (i.e., skip level) supervisors could train and when necessary to take the actions and managed the job demands of their subordinates (i.e., supervisors) which could lessen that frontline subordinates become victims of supervisors and stress releasing.

There are harmful consequences relative to abusive supervision which have been thoroughly documented. Past research has been performed relative to the antecedents of experienced supervisory abuse which have gained increased interest during the past several years; however, little is known about the influence of supervisors’ personality when viewing the prevalence of the apparent abuse. The study took a step to address this issue based on the moderated mediation model with the findings being positive when conscientiousness that was related to abusive supervision.

## 6. Boundaries and Future Opportunities

Our research has several limitations. First, the collection of the data occurred from different periods and could not confirm the causality. Furthermore, future research should apply a longitudinal design with a similar measure in each period to establish the causality.

Second, data were collected from the manufacturing sector. Results may be different if applied to another business sector. Different business sector should be taken into consideration for future research like the hospitality sector, banking sector, and telecommunications sector.

Third, data were collected from China (Asian country) having a different organizational structure. The results may not be applicable to the western context having different cultural values and business settings. The cross-cultural study should be recommended in the future for getting better results.

Finally, applying the COR theory, we found that emotional exhaustion is a strongest mediator in the relationship between abusive supervision and CWBs. Hence, another theoretical framework may help to explain the influential processes of abusive supervision. The present research treats counterproductive work behaviors (CWBs) as the dependent variable. Future research should find out how abusive supervision influences the reaction of subordinates.

## 7. Conclusions

Counterproductive work behavior is harmful when viewing the negative behavior of subordinates. The process also hinders the success and development of the organization. This study used the conservation of resources (COR) theory as discussed by Hobfoll [15] for job demand which was the inclusion of emotional exhaustion as an imperative mediator between abusive supervision and counterproductive work behavior. The moderated mediation model explained how emotional exhaustion and high job demand from the abusive leader lead to counterproductive work behavior in manufacturing in the country of China. The findings provided solid proof relative to the adverse consequences of abusive supervision by enlightening the effects of the unexplored mediator or moderator based on the relationship that existed between emotional exhaustion and CWB. Future constructs can be explored in depth to discover the motives of employee counterproductive work behavior.

## Figures and Tables

**Table 1:** Results of CFA for all exogenous and endogenous factors.

Model	*X* ^2^	*df*	TLI	CFI	RMSEA
The base line model (Four-factor model)	129.16	80	0.94	0.96	0.032
(Three-factor model-1:)	210.77	83	0.91	0.93	0.147
Abusive supervision and job demands combined					
(Three-factor model-2:)	201.54	83	0.93	0.91	0.118
Abusive supervision and emotional exhaustion combined					
(Three-factor model-3:)	185.29	83	0.82	0.85	0.193
Emotional exhaustion and CWB combined					
(Three-factor model-4:)	334.65	83	0.92	0.94	0.192
Job demands and emotional exhaustion combined					
(Two-factor model-1:)	928.64	85	0.88	0.76	0.233
Abusive supervision, Job demands and emotional exhaustion combined					
(Two-factor model-2:)	334.98	85	0.86	0.59	0.254
Abusive supervision and Job demands combined, CWB and emotional exhaustion					
(One-factor model-1:)	433.88	62	0.42	0.52	0.287

*N* = 350, Acronyms: CFA = Confirmatory Factor Analysis, RMSEA = Root Mean Square Error of Approximation, TLI = Tucker Lewis index, CFI = Comparative fit index.

**Table 2:** Mean, Correlation, Standard Deviation for all original variables.

	Variables	1	2	3	4	5	6	7	9	9	10	11
1	Education											
2	Age	−0.19 *										
3	Gender	0.08	0.05									
4	Tenure	0.17	0.08	0.05								
5	Organizations level	0.32 **	0.02 **	0.04	0.16 *							
6	PA	0.32 **	0.05	0.16 *	0.04	0.07	0.91					
7	NA	−0.17 *	0.07	−0.05	0.01	−0.04	0.12	0.94				
8	AS	−0.51	0.22 **	0.45	0.36	−0.21	−0.11	0.48	0.89			
9	JD	0.14 *	−0.16 *	0.21	0.3	0.12	−0.01	0.46	0.34	0.86		
10	EE	0.23	−0.09	0.34	0.01	−0.19	0.65	−0.03	0.22 **	0.08	0.90	
11	CWB	−0.15 *	0.12	0.14 *	0.19	0.01	0.12	0.16 *	0.13 **	0.22	0.18 ***	0.96
M		2.38	2.9	0.04	0.22	0.32	2.29	4.19	4.29	3.65	2.74	2.55
SD		5.61	6.52	0.89	0.96	0.51	1.21	4.54	6.52	1.13	4.76	0.18

*N* = 350; * p < 0.05, ** *p <* 0.01*,* *** *p* < 0.001; Bold values in diagonal are Cronbach’s alpha value of each variable. Acronyms: PA = Positive affect, NA = Negative Affect, AS = Abusive supervision, JD = job demand, EE = emotional exhaustion, CWB = counterproductive work behavior. M = Mean, SD = Standard Deviation.

**Table 3:** Results of hypotheses testing.

Variables	Emotional Exhaustion		CWB	
	Model 1	Model 2	Model 3	Model 4
**Control variables**				
Education	−0.09	−0.01	−0.05	−0.02
Age	−0.01	−0.19	−0.18	−0.18
Gender	−0.02	−0.03	−0.05	−0.04
Tenure	0.01	−0.01	−0.01	0.01
Organizational level	0.11	0.54	0.53 *	0.50
Positive affectivity	−0.04	−0.09	−0.14	0.12
Negative affectivity	0.23 **	0.28 **	0.35 **	0.29 **
**Independent variable**				
Abusive supervision	0.22 **	0.02	0.11 *	0.23
**Moderator**				
Job demands	0.03	0.24 **	0.18 **	0.18 **
**Interaction term**				
Abusive supervision × Job demands		0.29 **	0.12 **	0.13 **
**Mediator**				
Emotional exhaustion			0.27 **	0.18 **
*R^2^*	0.08	0.11	0.14	0.15
∆*R^2^*	0.02	0.03	0.01	0.04
*F*	3.76 ***	11.79 **	3.16 **	6.00 *
∆*F*	3.76 ***	8.03 **	3.16 **	2.84 **

*N* = 350; * *p <* 0.05 (two-tailed), ** *p* < 0.01 (two-tailed), *** *p* < 0.001 (two-tailed).

**Table 4:** The results of moderated path analysis.

Variables	AS→EE→CWB				
Moderator: Job Demands	Stage		Effect		
	First	Second	Direct	Indirect	Total
	*P_MX_*	*P_YM_*	*P_YX_*	(*P_MX_P_YM_*)	(*P_YX_ + P_MX_P_YM_*)
Simple path for high job demands	0.26 **	0.31 **	0.29 **	0.08 **	0.37 **
Simple path for low job demands	0.09 **	0.25 **	0.11 **	0.02	0.13 **
Differences	0.17 **	0.06 **	0.18 **	0.06 **	0.24 **

*N* = 350, * *p <* 0.05 (two-tailed), ** *p* < 0.01 (two-tailed); P_MX_ = Path from AS to EE; P_YM_ = Path from EE to CWB; P_YX_ = Path from AS to CWB. Low job demands refer to one standard deviation below the mean of job demands; High moderator refers to one standard deviation above the mean of job demands. Tests of differences between the indirect and total effects were based on bias-corrected confidence intervals derived from bootstrap estimates.
